# Casting a Wide Net: HIV Drug Resistance Monitoring in Pre-Exposure Prophylaxis Seroconverters in the Global Evaluation of Microbicide Sensitivity Project

**DOI:** 10.9745/GHSP-D-21-00122

**Published:** 2022-04-28

**Authors:** Lisa Levy, Jill M. Peterson, Lauren D. Kudrick, Bhavna Chohan, Everline Bosek, Irene Mukui, Mary Mugambi, Sarah Masyuko, Owen Mugurungi, Nonhlanhla Ndlovu, Imelda Mahaka, Megan Dunbar, Anita Hettema, Rudo A.P. Kuwengwa, Sindy Matse, Saiqa Mullick, Letitia Greener, Cara O'Connor, Diantha Pillay, Maria Fawzy, John W. Mellors, Urvi M. Parikh, the Global Evaluation of Microbicide Sensitivity (GEMS) project

**Affiliations:** a FHI 360, Washington, DC, USA.; b University of Pittsburgh, Pittsburgh, PA, USA.; c University of Washington, Seattle, WA, USA.; d Kenya Medical Research Institute, Nairobi, Kenya.; e Drugs for Neglected Diseases initiative, Nairobi, Kenya.; f National AIDS & STI Control Program, Nairobi, Kenya.; g Ministry of Health and Child Care of Zimbabwe, Harare, Zimbabwe.; h Pangaea Zimbabwe AIDS Trust, Harare, Zimbabwe.; i ICAP Coverage, Quality, and Impact Network, New York, NY, USA.; j Eswatini Ministry of Health, Mbabane, Eswatini.; k Wits Reproductive Health and HIV Institute, Faculty of Health Science, University of Witwatersrand, Johannesburg, South Africa.; l Population Services International, Johannesburg, South Africa.; m Anova Health Institute, Johannesburg, South Africa.; n International Partnership for Microbicides, Johannesburg, South Africa.; o Full GEMS Project Team listed at the end of the article.

## Abstract

**Background:**

Evidence of HIV drug resistance (HIVDR) in individuals using oral pre-exposure prophylaxis (PrEP) who acquire HIV is limited to clinical trials and case studies. More data are needed to understand the risk of HIVDR with oral PrEP during PrEP rollout. Mechanisms to collect these data vary, and are dependent on cost, scale of PrEP distribution, and in-country infrastructure for the identification, collection, and testing of samples from PrEP seroconverters.

**Methods:**

The Global Evaluation of Microbicide Sensitivity (GEMS) project, in collaboration with country stakeholders, initiated HIVDR monitoring among new HIV seroconverters with prior PrEP use in Eswatini, Kenya, South Africa, and Zimbabwe. Standalone protocols were developed to assess HIVDR among a national sample of PrEP users. In addition, HIVDR testing was incorporated into existing demonstration projects for key populations.

**Lessons learned:**

Countries are supportive of conducting a time-limited evaluation of HIVDR during the early stages of PrEP rollout. As PrEP rollout expands, the need for long-term HIVDR monitoring with PrEP will need to be balanced with maintaining national HIV drug resistance surveillance for pretreatment and acquired drug resistance. Laboratory capacity is a common obstacle to setting up a monitoring system.

**Conclusions:**

Establishing HIV resistance monitoring within PrEP programs is feasible. Approaches to drug resistance monitoring may evolve as the PrEP programs mature and expand. The methods and implementation support offered by GEMS assisted countries in developing methods to monitor for drug resistance that best fit their PrEP program needs and resources.

## Abstract (precis)

Global Evaluation of Microbicide Sensitivity projects in 4 countries demonstrated the feasibility of establishing an HIV drug resistance monitoring program for pre-exposure prophylaxis (PrEP). These projects will provide valuable information on seroconversions in the context of PrEP use and will serve to inform Ministries of Health and policy makers on the need for long-term surveillance approaches.

## Teaser

**Key Findings:**

- Monitoring for HIV drug resistance (HIVDR) within pre-exposure prophylaxis (PrEP) programs is feasible, and samples collected through these methods can be used to provide early indicators of the risk of HIVDR among PrEP users.
- Additional information beyond clinical trial data is needed on the risk of HIVDR in large-scale PrEP rollout programs, where HIV testing occurs less frequently and adherence support strategies are less robust.
- Laboratory capacity remains a major obstacle to establishing HIVDR monitoring systems in low- and middle-income countries.

**Key Implications:**

- Policy makers should consider short-term and long-term HIVDR monitoring needs to best preserve antiretrovirals for use in PrEP and antiretroviral therapy (ART) programs.
- By coordinating PrEP resistance monitoring with existing HIVDR surveillance, countries may be able to develop a common understanding of overall drug resistance and implications for national PrEP and ART programs.
- As new PrEP drugs and delivery methods, such as the vaginal ring and the long-acting injectable PrEP become available and as countries transition to alternative first-line ART regimens, more research and surveillance are needed to limit the risk of HIVDR.

## INTRODUCTION

Oral pre-exposure prophylaxis (PrEP) with tenofovir disoproxil fumarate and emtricitabine (TDF/FTC) substantially reduces the risk of HIV-1 acquisition when used daily as part of a combination HIV-1 prevention package.^1^ Approximately one-third of countries globally have approved TDF/FTC or TDF/lamivudine (3TC) for use as PrEP, with some offering PrEP through a national rollout program^2^ to all individuals at risk of HIV acquisition, and some through nongovernmental organizations or academic partner-led demonstration projects targeting priority and key populations including adolescent girls and young women, serodifferent couples, men who have sex with men, sex workers, and transgender people.^3^^–^^6^

PrEP is highly effective in preventing HIV aquisition^7^^,^^8^; however, infections can occur if PrEP is inadvertently initiated during the seronegative window of acute infection or with suboptimal adherence.^9^ PrEP individuals who seroconvert are at risk for developing HIV drug resistance (HIVDR). In randomized trials and open-label studies of TDF/FTC PrEP, 4.5% of participants who became HIV positive during the follow-up period had drug-resistant HIV-1 while 40% of individuals, retrospectively determined to have started PrEP while acutely infected by detecting viral RNA levels, had drug-resistant HIV-1.^8^^,^^10^^–^^15^ Several cases of seroconversion on PrEP despite high adherence have been reported in men who have sex with men whose partner(s) may have transmitted multidrug-resistant virus to them.^16^^–^^23^

While these data provide insight on the risk of HIVDR with PrEP use during clinical trials, the risk may differ in larger-scale PrEP rollout programs. During PrEP use outside of the controlled environment of a clinical trial, there may be diagnostic challenges including delayed detection of seroconversion and longer intervals between HIV tests, both of which could lengthen the time an individual may remain on PrEP after becoming HIV positive.^24^^,^^25^ In large-scale programmatic implementation, PrEP will likely be initiated without using nucleic acid tests to rule out acute infection. In addition, regular adherence to PrEP may fluctuate more than in clinical trials, when individuals had regular counseling and robust adherence support.^26^ Lastly, stock-outs of commodities could lead to PrEP interruption.^27^

Importantly, PrEP rollout is occurring in the context of rising global rates of transmitted HIVDR. According to the World Health Organization (WHO) 2021 HIVDR report, a majority of surveys performed between 2014–2020 reported pretreatment HIVDR above 10% (Box 1). The WHO recommends that countries switch to a dolutegravir-containing regimen for first-line antiretroviral therapy (ART) when a country reaches this 10% indicator.^28^ Additional modeling data reinforces the importance of ART programs avoiding a non-nucleoside reverse transcriptase inhibitor (NNRTI)-based first-line ART regimen in recent PrEP users who were diagnosed with HIV.^29^

BOX 1 **Pretreatment HIV Drug Resistance Definition**

Pretreatment drug resistance is resistance that is detected in individuals prior to initiating or reinitiating antiretroviral therapy (ART) (e.g., in women exposed to antiretroviral drugs for the prevention of mother-to-child-transmission of HIV, in people who have received pre-exposure prophylaxis, or in individuals reinitiating first-line ART after a period of treatment interruption without documented virologic failure).^28^

Therefore, it is essential for countries to monitor HIVDR in seroconversions on PrEP to preserve the effectiveness of both their PrEP and ART programs. The WHO Global HIV Drug Resistance Network (ResNet) developed global standards to monitor for HIVDR in PrEP programs and released them under a concept note titled, *HIV Drug Resistance Surveillance in Countries Scaling up Pre-Exposure Prophylaxes.*^30^ The concept note recommends that countries integrate a resistance monitoring surveillance strategy into their PrEP programs.

It is essential for countries to monitor HIVDR in seroconversions on PrEP to preserve the effectiveness of both their PrEP and ART programs.

To identify the risk of HIVDR within expanding PrEP programs, more data are needed in settings where PrEP is rolled out. To address this critical need, the President's Emergency Plan for AIDS Relief (PEPFAR)/U.S. Agency for International Development (USAID)-supported Global Evaluation of Microbicide Sensitivity (GEMS) project, in collaboration with country stakeholders, initiated HIVDR monitoring in Eswatini, Kenya, South Africa, and Zimbabwe, between 2018 and 2021. The data collected through these monitoring programs will be a valuable resource and will help to answer important public health questions on the risk and impact of HIVDR on PrEP programs. In this article, we present the process, methods, and implementation techniques that were used to establish HIVDR monitoring programs across 4 countries.^31^

## METHODS

The GEMS project used 2 methods to monitor for HIVDR in Eswatini, Kenya, South Africa, and Zimbabwe: (1) a standalone adaptable HIVDR monitoring protocol that enrolled individuals using PrEP that were receiving routine (nonresearch) services; and (2) incorporation of HIVDR into ongoing, established demonstration and pilot PrEP projects. Both methods used similar study procedures, implementation materials, data collection, and monitoring strategies.

### Adaptable HIVDR Monitoring Protocol

We developed an adaptable protocol template with a multisite cross-sectional study design to comprehensively assess HIVDR among seroconverters using TDF-containing PrEP with a goal of enrolling 50–300 participants per country within a study duration of 3 years (Supplement). The protocol included a primary objective to assess the frequency of HIVDR mutations among individuals using PrEP who test HIV positive after initiating PrEP and an exploratory objective to examine the relationship between HIVDR and PrEP adherence in individuals who seroconvert after starting PrEP. Inclusion criteria were kept broad and allowed enrollment for any current PrEP user, defined as an individual who has collected an initial or resupply of PrEP agents in the last 3 months independent of self-reported adherence and who is identified as HIV positive in accordance with the national HIV testing guidelines in their country.

Participants gave consent to participate in the study and to provide a blood sample through a streamlined, 1-page written informed consent form, available in English and the local language(s). The protocol template included optional additional requirements for age to comply with country-specific national PrEP guidelines.

### Incorporating HIVDR Into Ongoing PrEP Projects

GEMS also partnered with PrEP demonstration and pilot implementation projects to add resistance testing into existing Institutional Review Board/Ethics Committee (IRB/EC)-approved protocols through a protocol modification. These projects were identified by representatives on the GEMS steering committee comprised of experts and stakeholders from each of the countries GEMS worked in and those that expressed interest in collaboration. Participants enrolled in pilot projects, as per the applicable eligibility criteria, and were provided PrEP per protocol or national guidelines. The protocols and consent language allowed for blood collection for HIVDR testing at the time of seroconversion, and GEMS investigators were added into the protocol through a modification. These projects were not centrally managed by GEMS, but GEMS provided support for the HIVDR component through training, implementation materials, sample transport, collaboration with laboratories, and study monitoring.

### Study Procedures

Both methods involved a single visit where venipuncture blood was drawn and processed according to standard operating procedures, at the time of, or as soon as possible after, the initial HIV antibody positive rapid test(s) for seroconverters who had access to PrEP within the previous 3 months. Using a GEMS-provided kit, dried blood spots (DBS) were prepared and shipped at ambient temperature to the central HIVDR laboratory.

At clinics with access to daily transport to the testing laboratory, whole blood was collected for processing to plasma (Table 1).^32^^–^^34^ National or regional laboratories were identified and assessed for their proximity, accreditation standards, capability, and availability to receive and test HIVDR specimens. In-country testing occurred in Kenya and South Africa; samples from Eswatini and Zimbabwe were shipped to South Africa. Back-up laboratories were added to protocols, as allowed per applicable IRB/EC, for quality control purposes and to address testing difficulties or delays with the primary laboratory. A brief laboratory request form accompanied the blood specimen, which included the date of PrEP initiation, date of detected seroconversion, participant demographics, and self-reported adherence categories, encompassing adherent, somewhat adherent, and nonadherent options. Testing for resistance was planned in real-time to return results to participants; a separate DBS card for each sample was stored for batched testing of TDF drug levels at periodic intervals.

**TABLE 1.** Considerations for Collecting Blood Samples for HIV Drug Resistance Monitoring^32–34^

Sample Type	Storage	Transport and Shipment	Equipment	Training Needed	Blood Testing Capability
DBS	Ambient temperature storage for up to 14^a^ days; Long term storage in non-frost-free -20°C freezer or ≤-70°C freezer	DBS cards may be transported in an envelope to the lab at ambient temperature within 14 days	DBS cards, blood tube, and pipette to spot the cards	Minimal training required; clinical staff may have DBS experience with infant HIV testing	Five spots (per DBS card), limiting amount of blood available for testing; quality control or troubleshooting
Whole blood (for plasma)	Whole blood can be refrigerated prior to shipment (up to 24 hours) or immediately processed for plasma to be kept in ≤-70°C freezer for storage	Whole blood optimally shipped using ice packs; Plasma must be shipped on dry ice and stored at ≤-70°C	EDTA blood tube to collect whole blood (heparin not suitable); centrifuge needed to separate plasma from blood	Minimal training required for whole blood collection; additional laboratory training required for plasma preparation	Quantity of blood collected in tube allows for HIVDR, with leftover blood available for quality control or troubleshooting

Abbreviations: DBS, dried blood spot; EDTA, ethylenediaminetetraacetic acid; HIVDR, HIV drug resistance.

a The GEMS project aimed to complete sample transport within 5 days.

### Study Implementation Materials

Training slides, job aids, and fact sheets were all developed and compiled into a toolkit to support successful protocol implementation (Figure).^35^^–^^37^ All materials were developed by GEMS and underwent extensive iterative review with implementing partners, laboratory personnel, and country stakeholders to meet the needs of participating clinics, support participant understanding, and ensure specimen collection and testing were done according to specifications outlined in the protocol and standard operating procedures. The materials are available on the AVAC PrEP Watch website (https://www.prepwatch.org/gems/), as well as provided directly to implementing partners, either through trainings and meetings or included as part of the specimen collection kit that was distributed to clinics.

**FIGURE:**
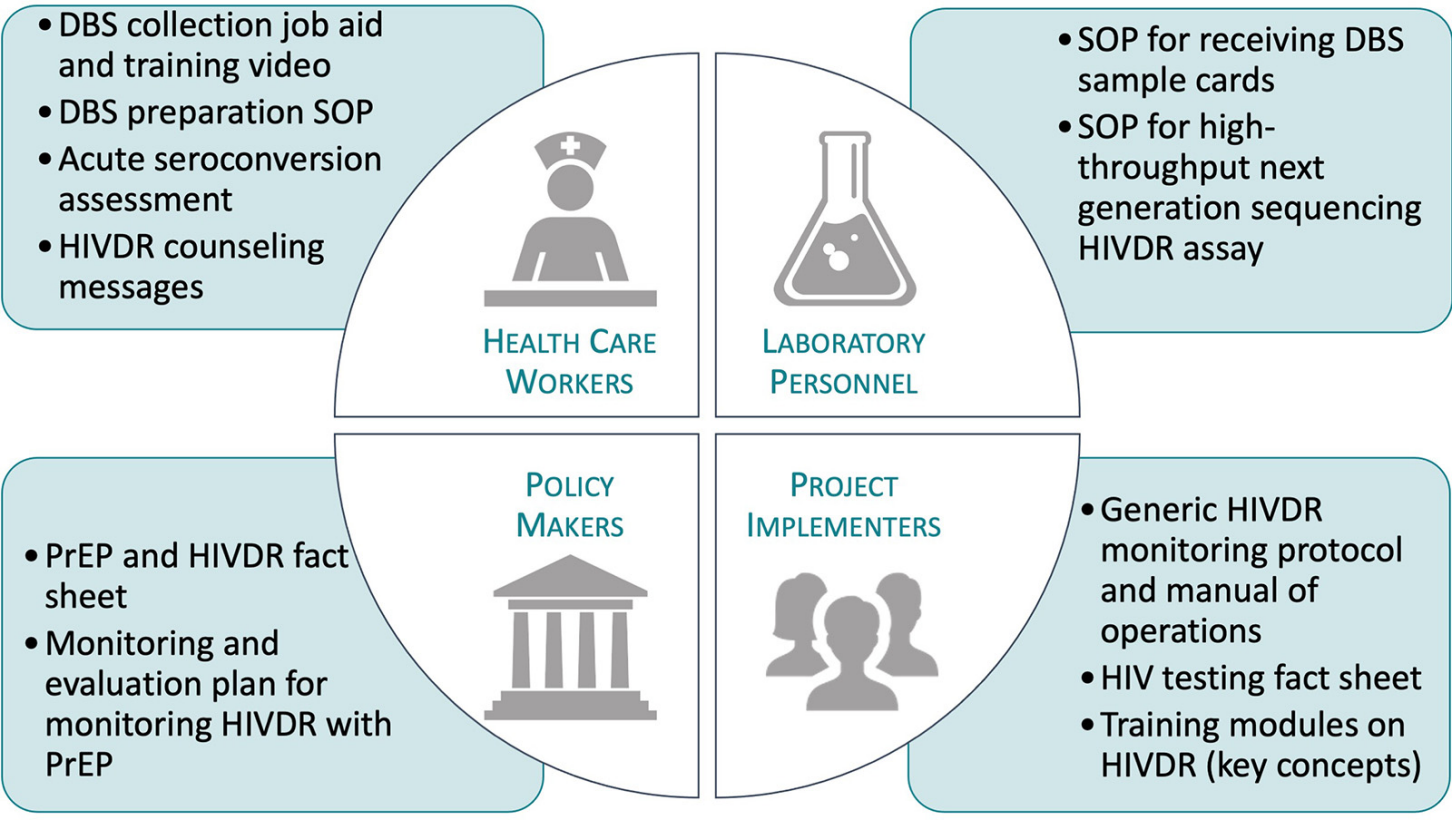
Global Evaluation of Microbicide Sensitivity HIV Drug Resistance Monitoring Toolkit^a^ Abbreviations: DBS, dried blood spot; HIVDR, HIV drug resistance; PrEP, pre-exposure prophylaxis; SOP, standard operating procedure. ^a^The Global Evaluation of Microbicide Sensitivity HIV Drug Resistance Monitoring Toolkit was developed to support implementation for pre-exposure prophylaxis resistance monitoring. The toolkit contains customizable materials for health care workers, laboratory personnel, policy makers, and project implementers including standard operating procedures, job aids, fact sheets, monitoring and evaluation plans, and training modules. The toolkit is available online at prepwatch.org/gems and gems.pitt.edu.

**Figure:**
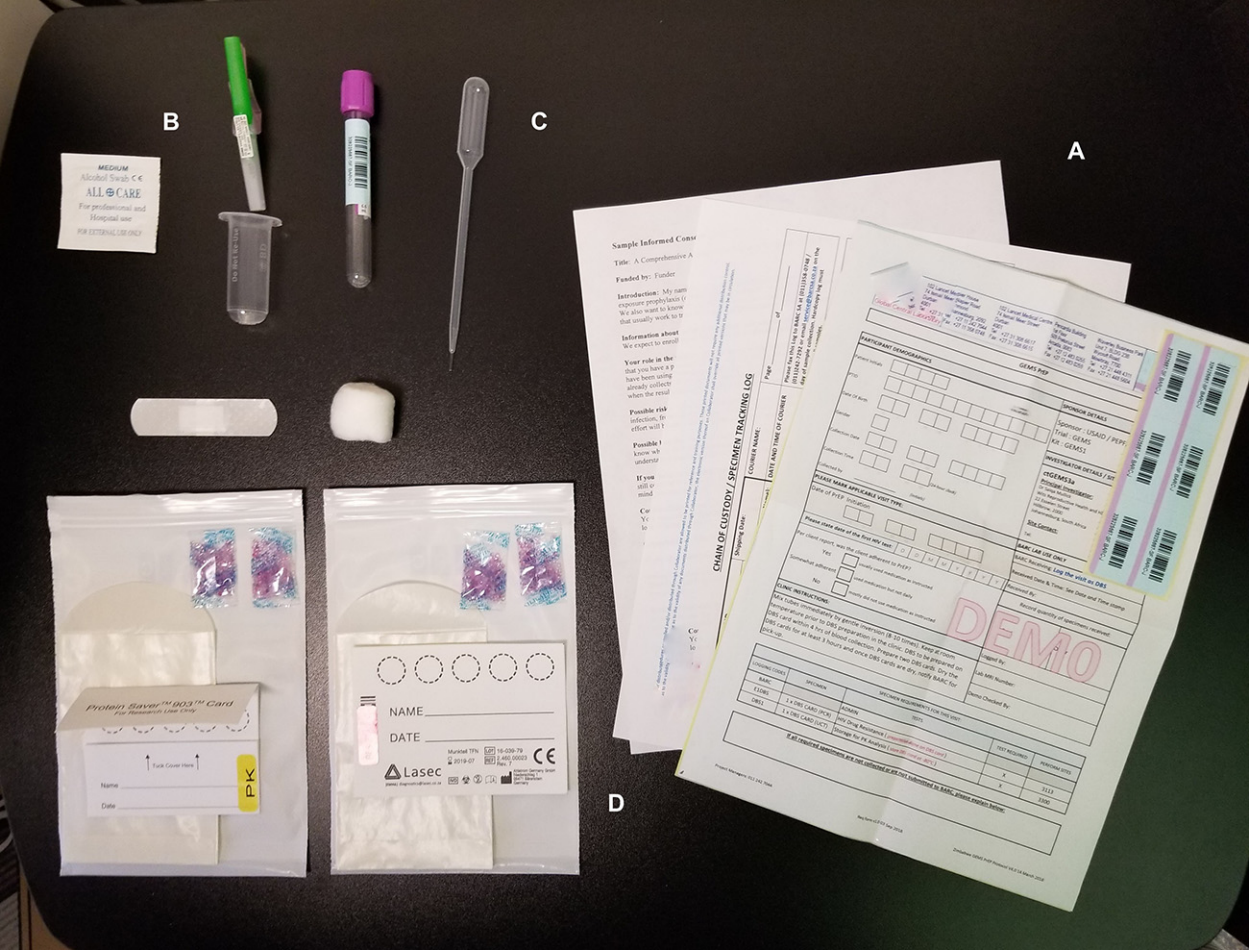
A Global Evaluation of Microbicide Sensitivity specimen collection kit was provided to pre-exposure prophylaxis (PrEP) delivery sites that included the following components: (A) Laboratory requisition form that includes date of PrEP initiation, date of detected seroconversion, client demographics, and self-reported adherence information; Chain of custody form for sample tracking; and Informed consent form to be signed by the client before sample collection. (B) Venipuncture blood collection materials including sterile wipe, ethylenediamine tetraacetic acid (EDTA) blood collection tube, needle, cotton wool, and plaster bandage; (C) Blood transfer materials including transfer pipette and 2 dried blood spot cards (one each for HIV drug resistance and drug-level testing); (D) Dried blood spot shipment and storage materials including desiccants, glassine envelopes, and sealable plastic bags. A shipping envelope with the address of the laboratory and prepaid postage was also provided. © 2018 Lauren Kudrick/University of Pittsburgh.

### Study Monitoring

Study implementation across all protocols was reviewed remotely, with a limited number of visits to PrEP clinics and testing laboratories to review procedures and ensure compliance with the International Council for Harmonisation Good Clinical Practice guidelines. Virtual meetings and in-person visits helped to identify challenges or bottlenecks and allowed for refresher training as needed. Common monitoring and evaluation indicators^38^ were established and routinely reviewed by the respective country-specific project teams. In addition, in-country project coordinators tracked and managed development and distribution of sample collection kits, number of seroconverters, number of samples collected and tested (using barcoded labels), and return of test results in coordination with the testing laboratories for each project. Some projects used a national database, while others tracked specimens through an internal system. Project coordinators communicated closely with testing laboratories to ensure the timely return of test results and contacted health clinics if tests were delayed or if laboratory requisition forms were not accurately completed. Study progress, successes, and challenges were presented to study investigators during routine meetings. When the study was conducted under a national protocol, presentations were also shared with the respective ministry of health (MOH) and HIV prevention-focused technical working groups.

## RESULTS

The GEMS project, in collaboration with country stakeholders, initiated MOH-led national monitoring of PrEP seroconverters for HIVDR in Eswatini, Kenya, and Zimbabwe, while drug resistance testing for seroconverters was incorporated into existing demonstration and pilot PrEP projects in South Africa involving key populations at risk of HIV. These countries were supportive of conducting time-limited evaluations of HIVDR during the early stages of PrEP rollout given the limited drug resistance data from seroconversions among individuals using PrEP. These projects demonstrated feasibility in establishing an HIVDR monitoring program, coordinating with providers to identify seroconverters, collecting a sample at the time of a first positive HIV test, and arranging shipment of specimens both within and outside the countries.^39^^,^^40^

These projects demonstrated feasibility in establishing an HIVDR monitoring program, coordinating with providers to identify seroconverters and collecting samples at the time of a first positive HIV test.

### Successful Protocol Adaptation

The national protocols for Eswatini, Kenya, and Zimbabwe were adapted from a customizable GEMS HIVDR protocol template (Supplement) and were chaired by local investigators and representatives from the country's MOH. GEMS project coordinators, along with partner organizations throughout all countries, facilitated protocol submission to their respective IRB/EC. Inclusion of HIVDR monitoring into pilot projects did not require substantive updates to existing protocols, as most already included blood collection for seroconverters. Protocol amendments were developed to include GEMS investigators and to allow for HIVDR testing if needed. The national protocols were developed by GEMS, and drafts were reviewed by study investigators, including representatives from the MOH, as applicable. All protocols required IRB/EC submission and approval, with timelines ranging from 3 months to 5 months (Table 2).

**TABLE 2.** HIVDR Monitoring Project Initiation by Country

Country	HIVDR Monitoring Approach	Implementing Partners	Timeline for IRB/EC Approvals^a^	Training Strategy	Laboratory Location and Sample Type
Eswatini	National seroconverter protocol, led by MOH	MOH, CHAI, private, and public national health facilities offering PrEP	3 months	Regional training approach (11 trainings/ 231 participants) and 5 add-on trainings (143 participants)	Johannesburg, South Africa; DBS and whole blood collected
Kenya	National seroconverter protocol, led by Kenyan investigators including MOH	MOH, CASCOs, POWER, Partners-Scale Up, PrIMA, PrIYA, LVCT Health, Jilinde, SWOP, CHAK, CHS, IRDO, PATH, APHIA, KEMRI Welcome Trust, Kilifi, NRHS, NOPE, I Choose Life, EGPAF, KENSHE, DPEP	3 months	Train-the-trainer approach (20 individual trainings conducted at county level and with individual partner organizations); additional training of PrEP service providers in the 10 highest incidence counties	Kisumu Kenya; DBS is primary method; however, plasma samples collected where possible, in case of DBS testing challenges
Zimbabwe	National seroconverter protocol, led by Zimbabwean investigators including MOHCC	MOHCC, PZAT, PSI, Zim-TTECH, CeSSHAR, OPHID, and national health clinics	4 months	Train-the-trainer approach (25 individual trainings conducted across project partners)	Johannesburg, South Africa; DBS is primary method; however, plasma samples collected where possible, in case of DBS testing challenges
South Africa	Individual demonstration projects providing PrEP to a limited number of health facilities	POWER, CHARISMA, Project PrEP Wits RHI/Unitaid, Wits RHI/Key Populations, Wits RHI/PrEP SMART	Approximately 4-5 months for various projects	Comprehensive approach; all sites trained through project partners (9 individual trainings conducted across project partners)	Johannesburg, South Africa; whole blood sample collected and sent to laboratory for any site with laboratory pick up capabilities within one day; DBS samples collected at all other clinics

Abbreviations: APHIA, AIDS, Population & Health Integrated Assistance; CASCO, County AIDS and Sexually Transmitted Infection Coordinator; CeSSHAR, Centre for Sexual Health and HIV AIDS Research Zimbabwe; CHAI, Clinton Health Access Initiative; CHAK, Christian Health Association of Kenya; CHARISMA, Community Health Clinical Model for Agency in Relationships and Safer Microbicide Adherence; CHS, Center for Health Solutions-Kenya; DBS, dried blood spot; DPEP, doxycycline post-exposure prophylaxis for prevention of sexually transmitted infections; EGPAF, Elizabeth Glaser Pediatric AIDS Foundation; HIVDR, HIV drug resistance; IRDO, Impact Research and Development Organization; KEMRI, Kenya Medical Research Institute; The KEN-SHE Study, KENya Single-dose HPV-vaccine Efficacy; MOH, Ministry of Health; MOHCC, Ministry of Health and Child Care; NOPE, National Organization of Peer Educators; NRHS, Nyanza Reproductive Health Society; OPHID, Organization for Public Health Interventions & Development; POWER, Prevention Options for Women Evaluation Research; PrEP, pre-exposure prophylaxis; PrIMA, PrEP Implementation for Mothers in Antenatal Care, PrIYA, PrEP Implementation in Young Women and Adolescents; PSI, Population Services International; PZAT, Pangaea Zimbabwe AIDS Trust; SWOP, Sex Workers Outreach Project; Wits RHI/Unitaid, Project PrEP Wits Reproductive Health and HIV Institute Wits RHI/PrEP SMART (Sequential multiple assignment randomized trial); Zim-TTECH, Zimbabwe Training, Technical Assistance and Education Center for Health.

a Time from initial protocol submission to final institutional review board (IRB)/ethics committee (EC) approval.

### Trainings and Kit Distribution

Once IRB/EC approvals were received, the study team, led by the country project coordinators, arranged and conducted in-person and online/webinar study trainings. Although the protocols were streamlined for simplicity of study procedures, health facilities required training on the informed consent process, sample collection, sample shipment, and quality control measures. These trainings included a rationale for HIVDR monitoring to justify the extra burden on study nurses and clinicians, as well as to better inform potential participants about the benefits of their participation. Teams were also trained to ensure participant safety and confidentiality; for example, using a coded number to identify samples and data collection forms and ensuring all study-related information was stored securely at the health care facilities. Sample training slides were adapted for each protocol.^35^ Trainings typically were scheduled for approximately 1–2 hours and occurred centrally in a “train-the-trainer” approach to extend the reach to all facilities taking part in the study. Often the training for HIVDR testing would occur as a component of a larger PrEP training, organized by study partners or the MOH. GEMS project coordinators recognized the need for additional trainings to maximize awareness; however, the train-the-trainer approach did not always trickle down to individual clinics. For countries implementing a national protocol with a large number of PrEP clinics geographically spread across the country, project teams would prioritize additional trainings at sentinel sites—those with a high volume of PrEP users and/or located in high HIV incidence areas.

At the time of training or soon thereafter, project teams distributed the forms required for participants to give consent and sample collection kits with materials to collect a blood specimen and ship to the laboratory. These were distributed to (1) implementing partners, which in turn would distribute kits to individual clinics; (2) directly to clinics with high volumes of PrEP users; and/or (3) to centrally located regional or county offices. Project coordinators closely tracked kit distribution, replacing used kits or kits that included expired components such as blood tubes or DBS cards. Due to the location and high volume of facilities providing PrEP in both Kenya and Zimbabwe, the study teams opted to provide county/province level offices with blood collection kits and supplies. The individual health facility then contacted the country/province office when a seroconversion occurred and requested a blood collection kit, which would typically arrive within 1 day.

### Study Implementation Toolkit

A toolkit of study implementation materials was developed for facilities that included a DBS collection job aid, acute seroconversion assessment, and further study-specific instructions for specimen collection, shipment, laboratory testing notifications, and results communication.^36^^,^^37^ As HIV seroconversions among PrEP users were expected to be rare, health facilities were encouraged to post the job aids on clinic walls as a reminder to collect a blood sample in the event of an HIV-positive test. Beyond costs of the training, printing job aids/flow charts, shipment of samples, and kit procurement, no additional funding or reimbursement, unless already included as part of the pilot project, was provided to health facilities or study participants.

### GEMS Implementation

Altogether, GEMS partnered with 41 organizations, conducted more than 85 trainings, and distributed approximately 800 sample collection kits. This provided coverage for individuals accessing PrEP across all regions, counties, and provinces in Eswatini, Kenya, and Zimbabwe; as well as the individual clinics participating in pilot projects in South Africa. GEMS-partnered projects provided a system to collect a blood sample on any seroconverter identified among the estimated 72,000 “cumulative PrEP users” as of 2020 in Kenya,^41^ 25,000 in Zimbabwe,^42^ and 9,500 in Eswatini^43^ (Box 2).^44^ In South Africa, this coverage included all 7,500 PrEP users participating in a GEMS-partnered pilot project. Across all countries, specimen shipment was arranged using national transportation systems or, in limited cases, collected directly from the project coordinator (or designee) to ensure samples reached the testing laboratory within the required timeframes to ensure the quality of the sample. Close communication between the project coordinators and testing laboratories ensured test results were returned and that delays in testing were communicated to the participating clinic.

BOX 2 **Cumulative Pre-Exposure Prophylaxis (PrEP) Users**

“Cumulative PrEP users” is a PrEP Watch indicator to measure the cumulative count of those who have ever used PrEP in their lifetimes, which includes numbers reported from demonstration projects, U.S. President's Plan for Emergency AIDS Relief dashboard, and program/country reports where available.^44^

## DISCUSSION

Most of what we currently know about HIVDR and PrEP use is based on analyses from clinical trials and demonstration projects, where HIV testing occurred frequently and which included robust adherence support strategies. Initial analyses of results from GEMS-supported studies found low rates of seroconversion overall. However, HIVDR related to PrEP was identified, demonstrating the importance of monitoring to better understand the risk of resistance with PrEP in an implementation setting and the underlying pretreatment resistance rates within countries.^31^^,^^40^ GEMS-partnered monitoring projects focused on time-limited approaches of assessing HIVDR risk through independent research protocols and pilot projects. Both strategies included training for clinics, informed consent procedures, and were dependent on access to an in-country or regional laboratory, which allowed for shipment feasibility (based on sample type) and timeliness of returning test results. The advantages of using a research protocol approach, include a discrete timeline for data collection, analysis, and review of results, resulting in a natural time for policy review and decision making at the conclusion of the study. Partnering with an established project also allowed for cost sharing and using existing facilities and specimen shipment infrastructure.

HIVDR related to PrEP was identified, demonstrating the importance of monitoring to better understand the risk of resistance with PrEP in an implementation setting and the underlying pretreatment resistance rates within countries.

It will be important for MOHs and implementing partners to evaluate the need for and possibility to expand to long-term monitoring options, such as incorporating testing strategies into national guidelines or periodic surveillance efforts (i.e., existing HIVDR surveillance system for populations starting ART [pretreatment HIVDR] or in populations receiving ART [acquired HIVDR]). A clear advantage to adding HIVDR for PrEP into an ongoing surveillance strategy is the ability to leverage existing sample collection, testing, and reporting systems. By coordinating surveillance techniques, countries may be able to develop a common understanding of overall drug resistance and implications for country PrEP and ART programs. Disadvantages include having sufficient numbers of samples collected from PrEP seroconverters if these are collected as part of PDR surveillance and the ability to disaggregate PrEP-specific HIVDR data from overall PDR data. With these long-term options, policy makers would need to consider the scale of implementation and whether testing would be offered within all health care facilities providing PrEP, alternative PrEP delivery sites, or in a subset of facilities based on criteria such as regional HIV prevalence, high volume facilities, or key populations of interest.

As PrEP programs expand globally, monitoring for HIVDR is an important tool in the evaluation toolbox used to measure programmatic success, providing information on potential challenges with PrEP adherence and/or HIV testing strategies. Furthermore, it may help to identify any potential impact on ART programs, supporting decisions on first-line treatment considerations for HIV-positive individuals who had previously used PrEP.^45^ Lastly, it may provide more information on potential shifts in PDR rates within a given country. As new PrEP drugs and delivery methods, such as the monthly dapivirine vaginal ring and the cabotegravir long-acting injectable, become available on the global market and as countries transition to alternative first-line ART regimens in the same drug classes as PrEP agents, more research and surveillance will be needed to monitor and limit the risk of HIVDR.^46^^,^^47^ Systems developed to monitor for HIVDR with PrEP use must be adaptable to these new PrEP agents as drug resistance risk and profiles may change.

Monitoring for HIVDR is an important tool in the evaluation toolbox used to measure programmatic success, providing information on potential challenges with PrEP adherence and/or HIV testing strategies.

### Challenges With Establishing HIVDR Monitoring Programs

An immediate challenge faced across all GEMS HIVDR monitoring projects was access to an accredited laboratory for testing. In Kenya, at the time of this project, there was 1 WHO-accredited laboratory for HIVDR testing on DBS and plasma samples in Kisumu; however, the National HIV Reference Laboratory is now undergoing WHO accreditation for HIVDR. Ongoing challenges with procuring reagents led to delays in testing and without an alternative accredited lab, the delays could not be avoided. Eswatini and Zimbabwe were without an accredited lab, necessitating all samples be transferred to a laboratory in South Africa, which tested samples from Eswatini, South Africa, and Zimbabwe. While plasma is the “gold standard” for HIVDR testing, DBS sample collection allowed for temporary specimen storage at ambient temperature, without specialized transport requirements. However, there is some evidence that PrEP use may lower initial viral loads, which impacts the ability to successfully perform an HIVDR test with DBS.^25^^,^^34^ Laboratory capacity remains a major obstacle to establishing monitoring systems in low- and middle-income countries. The *WHO Global Action Plan on HIV Drug Resistance* includes laboratory capacity to strengthen HIVDR testing as a strategic objective.^48^ In addition, new HIVDR testing assays are being developed to lower the cost, increase throughput, and potentially allow for point-of-care testing.^49^

### Limitations

An important limitation throughout these HIVDR monitoring projects is the impact of missing samples from unknown seroconverters when an individual never returns to retest or those lost to follow-up. National or project databases may not accurately document seroconversions, and HIVDR monitoring projects can only track sample collection and testing from identified and reported seroconversions. Additionally, known seroconverters may not give consent to provide a blood sample or may be lost to follow-up if they are immediately referred to the ART provider to initiate treatment. All these scenarios impact our ability to know the total number of seroconversions with HIVDR that may have occurred in a PrEP program.

Lastly, while implementing a national protocol allows for increased sample collection across diverse populations; providing training and establishing oversite across all public facilities that offer PrEP is difficult to establish and maintain. As PrEP rollout continued to expand in these countries over the course of GEMS implementation, new PrEP delivery sites were added rapidly, and the project teams had to react quickly to ensure new partners and clinics were trained on the study procedures and prepared to collect samples if a new seroconversion should occur.

## CONCLUSION

In the context of rising transmitted HIVDR rates in low- and middle-income countries, the risk of emerging HIVDR despite PrEP use should be monitored and addressed proactively. When developing a monitoring program, it is important to ensure early involvement with stakeholders, identify an efficient mechanism to collect samples and feed results back to policy makers, and provide ongoing training and technical support to implementing facilities. Investing in laboratory capacity, including infrastructure, equipment, standardized assays, and personnel training, should be a critical component of national strategies to support widespread implementation of HIVDR projects.

**Global Evaluation of Microbicide Sensitivity (GEMS) Team Authors:** Leadership Team: John W. Mellors, MD, Project Director, University of Pittsburgh School of Medicine; Urvi M. Parikh, PhD, Project Co-Director, University of Pittsburgh School of Medicine; Lauren D. Kudrick, MA, MEd, Project Administrator, University of Pittsburgh School of Medicine. Laboratory Team: Kevin McCormick, PhD, Research Scientist, University of Pittsburgh School of Medicine; Kerri Penrose, MS, Research Scientist, University of Pittsburgh School of Medicine; Amy Heaps, MS, Research Scientist, University of Pittsburgh School of Medicine; Barbra Richardson, PhD, Statistician, University of Washington; Uma Chandran, PhD, Bioinformatics, University of Pittsburgh Department of Biomedical Informatics; Rahil Sethi, MS, Bioinformatics, University of Pittsburgh Department of Biomedical Informatics. Modeling Team: Andrew Phillips, PhD, Lead Modeler, University College London; Fumiyo Nakagawa, PhD, Modeling Scientist, University College London; Valentina Cambiano, PhD, Modeling Scientist, University College London. Policy Team: Lisa Levy, MPH, Policy Team Lead, FHI 360; Kristine Torjesen, MD, MPH, Policy and Evaluation Advisor, FHI 360; Irina Yacobson, MD, Policy Specialist, FHI 360; Rick Homan, PhD, Health Economist, FHI 360; Maria Fawzy, MHA, Research Manager, FHI 360; Jill M. Peterson, MPP, Monitoring and Evaluation Director, FHI 360. Rollout Team: Carole Wallis, PhD, Senior Technical Advisor, BARC Laboratory; Bhavna Chohan, PhD, Kenya Country Coordinator, KEMRI/University of Washington; Everline Bosek, MSc, Kenya Program Manager, University of Washington – Kenya; Megan Dunbar, DrPH, Zimbabwe Country Coordinator, Consultant; Imelda Mahaka, BS, Zimbabwe Team Liaison, PZAT; Nonhlanhla Ndlovu, MA, Zimbabwe Program Manager, PZAT; Anita Hettema, MPH, Eswatini Country Coordinator, FHI 360. United States Agency for International Development: Delivette Castor, PhD, Columbia University; Lee Claypool, PhD, USAID; Lee Sims, MSc, MEng, USAID; Shannon Allen, PhD, USAID; Antoinette Nelson, PhD, USAID.

## Supplementary Material

GHSP-D-21-00122-supplement.pdf

## Notes

**Peer Reviewed**

**First published online:** April 18, 2022.
