# Supplementary material for: Casting a Wide Net: HIV Drug Resistance Monitoring in Pre-Exposure Prophylaxis Seroconverters in the Global Evaluation of Microbicide Sensitivity Project
Source: Glob Health Sci Pract. 2022 Apr 28;10(2):e2100122. doi: 10.9745/GHSP-D-21-00122 (PMC9053149; doi:10.9745/GHSP-D-21-00122)
Supplement: GHSP-D-21-00122-supplement.pdf [file GHSP-D-21-00122-supplement.pdf]

Supplement to: Levy L, Peterson J, Kudrick, et al. Casting a wide net: HIV drug resistance monitoring in pre-exposure prophylaxis seroconverters in the Global Evaluation of Microbicide Sensitivity Project. Glob Health Sci Pract. 2022;10(2): e2100122. https://doi.org/10.9745/GHSP-D-21-00122

A Comprehensive Monitoring Plan to Assess the Risk of HIV Drug Resistance among PrEP Users

Funding Agencies:

Protocol Chair:

Version X.X

DD MMM YYYY

Table of Contents

1. PROTOCOL SUMMARY 5
3. KEY ROLES 6
4. INTRODUCTION AND BACKGROUND 6
5. RATIONALE FOR STUDY DESIGN 8
6. STUDY OBJECTIVES 9
7. STUDY DESIGN AND METHODOLOGY 9
8. STUDY PROCEDURES 11
9. STATISTICAL CONSIDERATIONS 14
10. ETHICAL CONSIDERATIONS 14
11. DATA ANALYSIS 15
12. PUBLICATION POLICY AND RESULT DISSEMINATION 15
13. REFERENCES 16

ABBREVIATIONS

AGYW	Adolescent Girls and Young Women
AHI	Acute HIV Infection
ART	Antiretroviral Treatment
ARV	Antiretroviral
CDC	Centers for Disease Control and Prevention
DBS	Dried Blood Spot
DR	Drug Resistance
FSW	Female Sex Worker
GEMS	Global Evaluation for Microbicide Sensitivity
HIV	Human Immunodeficiency Virus
MOH	Ministry of Health
MSM	Men who have sex with men
PK	Pharmacokinetics
PrEP	Pre-exposure Prophylaxis
RNA	Ribonucleic Acid
SOP	Standard Operating Procedure
TDF/FTC	Tenofovir disoproxil fumarate/ Emtricitabine
TDF/3TC	TDF/lamivudine
TDR	Transmitted drug resistance
TFV	Tenofovir
WHO	World Health Organization

PROTOCOL INVESTIGATORS

Primary Investigator

[Name]
[Organization]
[Address]
[Phone]
[Email]

Co-Investigators

[Name]
[Organization]
[Address]
[Phone]
[Email]

[Name]
[Organization]
[Address]
[Phone]
[Email]

[Name]
[Organization]
[Address]
[Phone]
[Email]

Supplement to: Levy L, Peterson J, Kudrick, et al. Casting a wide net: HIV drug resistance monitoring in pre-exposure prophylaxis seroconverters in the Global Evaluation of Microbicide Sensitivity Project. Glob Health Sci Pract. 2022;10(2): e2100122. https://doi.org/10.9745/GHSP-D-21-00122

1. PROTOCOL SUMMARY

**Short Title:**	An assessment of the Risk of HIV Drug Resistance among PrEP Users
**Funder:**	[insert funding source]
**Protocol Chair:**	[insert name]
**Sample Size:**	Approximately [insert sample size] participants
**Client Population:**	Clients who acquire HIV-1 infection while using TDF, TDF/FTC or TDF/3TC for PrEP
**Study Design:**	Cross-sectional/Surveillance
**Study Duration:**	One time visit per participant with enrollment continuing until [MM/YYYY] or [insert sample size] participants are enrolled
**Study Methods:**	PrEP clients who have a reactive HIV test will have blood drawn to assess HIV drug resistance. The blood sample will be collected only once for each seroconverter, at the time of the first reactive HIV test result.
**Primary Objective:**	To assess the frequency of HIV-1 drug resistance mutations among PrEP clients who test HIV-positive after initiating PrEP
**Primary Endpoint:**	HIV-1 drug resistance mutations among seroconverters on PrEP
**Exploratory Objectives:**	1. To explore the relationship between HIV drug resistance and PrEP adherence in individuals who seroconvert on PrEP<br><br>2. To assess drug levels at time of seroconversion
**Exploratory Endpoints:**	1. Individual self-reported adherence or factors that may be associated with HIV drug resistance<br><br>2. Proportion of seroconverters with drug resistance that have detectable drug levels in blood

3. KEY ROLES

Funding Agencies: [insert name/address]

Laboratories: [insert name/address]
[insert back-up laboratory name/address, as applicable]

Data Center: [insert name/address]

Study Operations: [insert name/address]

4. INTRODUCTION AND BACKGROUND

Primary HIV prevention is an important component of efforts towards UNAIDS goal of elimination of HIV by 2030[1]. High incidence of HIV infection has been reported among high risk populations such as men who have sex with men (MSM), female sex workers (FSWs), HIV serodiscordant couples, and adolescent girls and young women (AGYW)[2]. Interventions to prevent HIV infection include mutual monogamy, male circumcision, consistent condom use, and antiretrovirals (ARVs) as pre-exposure prophylaxis (PrEP).

The World Health Organization (WHO) recommends the use of PrEP with daily oral emtricitabine plus tenofovir disoproxil fumarate (FTC/TDF) or Lamivudine (3TC) plus TDF as a prevention strategy for HIV uninfected individuals at substantial risk of HIV[3]. PrEP has been shown to substantially reduce the risk of HIV acquisition, especially when used in combination with other behavioral preventive methods[3]. PrEP efficacy trials in different populations have reported an HIV seroconversion incidence ranging from 0.3%[4] to 6.5%[5] [see Table 1]. In the Partners PrEP study, TDF/FTC provided 75% protection, while use of tenofovir alone offered 67% protection[6]. Although some PrEP users may still acquire HIV infection, protection against infection increased as adherence increased in TDF/FTC PrEP efficacy trials[7,8].

Table 1
HIV Incidence in PrEP Efficacy Trials

Study	Population[a]	TDF/FTC Arm Only	
		# Enrolled	# Infected
**FEM-PrEP[9]**	Women	1062	33
**iPrEX[10]**	MSM	1252	36
**TDF2[11]**	HS Men and Women	611	9
**Partners PrEP[6]**	HS Discordant Couples	1579	21
**VOICE[12]**	Women	1003	61
**PROUD[13]**	MSM	544	20
**IPERGAY[5]**	MSM	31	2
**USA DEMO Project[4]**	MSM/TW	557	2
**TOTAL**		**6638**	**184**

[a]HS = heterosexual; MSM = men who have sex with men; TW = transgendered women

Factors linked to risk of HIV seroconversion include social-behavioral, adherence and possible infection with TDF resistant strains[14]. The potential for overlapping resistance and cross-resistance between ARVs used for prevention and for treatment is an important concern for the large-scale roll-out of PrEP. The major factors influencing the selection or acquisition of resistance during PrEP use include the level and duration of ARV exposure during unrecognized acute infection, the occurrence of breakthrough infection due to suboptimal drug levels, and the prevalence of transmitted drug resistance (TDR) in the regions where PrEP roll-out is planned.

In addition to providing efficacy data, the TDF/FTC PrEP trials also revealed that HIV drug resistance is infrequently selected if PrEP is successfully started before HIV-1 infection has occurred and is more likely to occur when PrEP is inadvertently started during undiagnosed acute infection [see Table 2][15]. Though TDF/FTC rather than TDF/3TC PrEP was used in most of these trials, FTC and 3TC have similar chemical structures and, therefore, mutations in HIV at positions M184V/I or K65R decrease viral susceptibility to both ARVs. TDF susceptibility is also reduced with the K65R mutation, but TDF remains effective in the presence of M184V/I mutations[16].

Mathematical modeling suggests that the number of HIV-1 infections averted by the use of PrEP exceeds the increase in drug-resistant infections that could occur from PrEP [14,15]. Clinical identification of acute HIV infection will be an important component of PrEP programs to prevent development of drug resistance. However, acute infection is often either overlooked or misdiagnosed at outpatient care settings[17-19].

Table 2.
HIV-1 resistance to tenofovir or FTC in oral TDF/FTC PrEP trials[15]

Study	Number of Seroconverters in TDF/FTC Arm	TFV Resistance		FTC Resistance	
		Standard	Sensitive	Standard	Sensitive
Infection After Enrollment					
FEM-PrEP	33	0	0	4	1
iPrEX	36	0	0	0	2
TDF2	9	0	0	0	0
Partners PrEP	21	0	1	0	5
VOICE	61	0	0	1	2
TOTAL	160	**0 (0%)**	1 (0.6%)	**5 (3%)**	10 (6%)
Acute Infection at Enrollment					
FEM-PrEP	1	0	-	0	-
iPrEX	2	0	-	2	-
TDF2	1	1	-	1	-
Partners PrEP	4	0	-	2	-
VOICE	9	0	-	2	-
TOTAL	17	**1 (6%)**	-	**7 (41%)**	-

Currently, WHO recommends immediate initiation of antiretroviral therapy (ART) for all individuals diagnosed as HIV infected regardless of WHO clinical stage or CD4 count[3]. Early initiation of ART has several benefits that include fewer events of severe HIV morbidity and disease progression, improved uptake and initial linkage to care, better immune recovery and reduction in HIV transmission to negative partners[20]. In line with these guidelines, individuals who seroconvert while on PrEP will be advised to initiate ART immediately. However, it is uncertain if the failed PrEP prevention method would be associated with blunted response to ART due to the presence of drug resistant HIV strains.

Therefore, as countries implement national PrEP programs, it is important to assess individuals who seroconvert to determine the frequency of those seroconverters with HIV drug resistance mutations. These data will inform national PrEP programs and can help to ensure long-term effectiveness of both PrEP and ART.

5. RATIONALE FOR STUDY DESIGN

The primary objective of the study is to assess the frequency of HIV drug resistance with PrEP use. The potential for overlapping HIV drug resistance and cross-resistance between ARVs used for prevention and for treatment is an important global concern for the large-scale implementation of PrEP. However, there is currently a paucity of data on the risk of HIV drug resistance in the context of PrEP, all of which is based on limited data from completed PrEP clinical trials and not from real-world PrEP implementation. These clinical trials differed in many ways from current strategies for PrEP roll-out, including different intervals for HIV testing and adherence support strategies.

Supplement to: Levy L, Peterson J, Kudrick, et al. Casting a wide net: HIV drug resistance monitoring in pre-exposure prophylaxis seroconverters in the Global Evaluation of Microbicide Sensitivity Project. Glob Health Sci Pract. 2022;10(2): e2100122. https://doi.org/10.9745/GHSP-D-21-00122

This study is designed to measure the frequency of HIV drug resistance through a cross-sectional, surveillance method. This design allows for a one-time measurement of drug resistance following seroconversion on PrEP, limiting clinic and participant burden. Results will provide an overall prevalence of drug resistance in clients that seroconverted while taking PrEP. These data may help assess the quality of the PrEP program. A high rate of observed resistance may indicate changes needed to the timing of PrEP provision or improved diagnosis of acute infection. A low rate of observed resistance with a high rate of infection may indicate adherence issues. The specific mutations observed may indicate if resistance is predominantly transmitted or potentially unrelated to PrEP use. This drug resistance surveillance technique will also contribute to local capacity building to incorporate pre-treatment, and acquired drug resistance surveillance as per WHO recommendations.

Data from this study will inform the global community on the risk of HIV drug resistance on PrEP seroconverters. It will importantly inform PrEP implementation programs, by providing recommendations on HIV testing frequencies, and PrEP adherence strategies. Ultimately, it will help to ensure the long-term effectiveness of both PrEP and ART.

6. STUDY OBJECTIVES

Primary Objective:

To assess the frequency of HIV-1 drug resistance mutations among PrEP clients who test HIV-positive after initiating PrEP

Primary Endpoint:

HIV-1 drug resistance mutations among seroconverters on PrEP

Exploratory Objectives:

1. To explore the relationship between HIV drug resistance and PrEP adherence in clients who seroconvert on PrEP
2. To assess drug levels at time of seroconversion

Exploratory Endpoints:

1. Individual self-reported adherence or factors that may be associated with HIV drug resistance
2. Proportion of seroconverters with drug resistance that have detectable drug levels in blood

7. STUDY DESIGN AND METHODOLOGY

Study design

Multi-site, cross-sectional study, in facilities providing PrEP. The study will provide an estimate of the frequency of ARV resistance in the population of PrEP users who test HIV positive after initiating PrEP. Descriptive demographic characteristics, including gender and age, as well as adherence factors associated with HIV seroconversion, will be collected.

Throughout the study period, PrEP users who acquire HIV-1 will be provided information about this study by healthcare providers. If the PrEP user consents, the client will have blood drawn for preparing a dried blood spot (DBS) sample or a plasma sample for HIV drug resistance testing and pharmacokinetic (PK) testing. Procedures (detailed in Section 9) are scheduled to all be completed at the visit that seroconversion is identified. However, if for some reason procedures are not completed on the day of seroconversion, clients will be asked to return to the clinic to complete the procedure.

Study area description

The study will be conducted at facilities providing PrEP, with capacity for sample collection.

Study population

PrEP users who present for any visit and are identified as HIV infected during that visit will be potentially eligible for participation in the study, if they meet the inclusion criteria listed below.

<u>Inclusion criteria</u>

1. Current PrEP user - defined as an individual who has collected an initial supply of PrEP agents or a resupply of PrEP agents in the last three months, independent of self-reported adherence
2. Identified as HIV infected, as per the HIV Testing Algorithm in National Guidelines
3. Willing to participate in the study and provide a blood sample

Sample size and power calculations

The target number of PrEP initiators during the study period is [insert]; however, as additional sites rollout PrEP, the sample size may increase. The *sample size and power calculations* section below, detail assumptions of the HIV incidence rate that would lead to approximately [insert] PrEP seroconverters during the study period.

The primary objective will be measured via the proportion of HIV-infected individuals with evidence of drug resistance. Assuming a population of approximately [insert] PrEP users, an HIV incidence rate of [insert] per 100 person years, and a 10% refusal rate, the study should be able to enroll up to [insert] seroconverters over [insert] years. The proportion of those with drug resistance is expected to be low and less than [insert]% of the [insert] seroconverters.

As a means to characterize the statistical properties of this study Table 3 below presents the probability of observing zero, one or more and two or more events among 100 participants given a true event rate [*note: Tables below are illustrative of 100 seroconverters; will need to be adapted for varying sample size*]. For example, if the true rate of resistance is 10%, then the probability we will see 1 or more cases of resistance is 99%.

Table 3
Analysis of Resistance Frequency

"True" Event Rate	P (0 events | n=100)	P (≥1 events | n=100)	P (≥2 events | n=100)
1%	0.37	0.26	0.08
5%	<0.01	0.96	0.88
10%	<0.01	>0.99	>0.99

An alternative way of describing the statistical properties of the study design is in terms of the 95% confidence interval (95% CI) for the true rate based on the observed data. Table 4 below shows the exact 2-sided 95% CIs for the probability of an event based on a particular observed rate. If none of the 100 participants has resistant virus, the 95% exact 2-sided upper confidence bound for the true rate of resistance is 3.6%. Similarly, if 10 of the 100 participants have resistant virus, the 95% exact 2-sided lower confidence bound for the true rate of resistance is 4.9% and the upper bound is 17.6%.

Table 4
Precision of Exact 2-sided 95% CIs for Observed Event Rates

Observed Event Rate	Exact 2-sided 95% CI (n=50)
20/100 (20%)	12.7%, 29.2%
15/100 (15%)	8.7%, 23.5%
10/100 (10%)	4.9%, 17.6%
5/100 (5%)	1.6%, 11.3%
1/100 (1%)	<0.1%, 5.5%
0/100 (0%)	0%, 3.6%

8. STUDY PROCEDURES

This is a cross-sectional study conducted with PrEP clients who acquire HIV infection. Accrual will continue until approximately [insert] HIV-positive participants have been enrolled or until [MM/YYYY]. Blood collection is expected to be completed at one visit. Figure 1 presents the operational flow that will be repeated each time a PrEP client returns for an HIV test. Samples will be shipped to the [insert] laboratory and the [insert] laboratory for drug resistance testing and drug level testing, respectively. [Add back-up laboratories as needed].

9a. Recruitment

Consecutive PrEP users who seroconvert will be recruited until the sample size is reached, or the study closes. PrEP users identified as HIV infected by the healthcare worker will be informed about the study and referred to the designated study coordinator, which may be a Nurse, Clinical officer, or Medical officer. The study coordinator will inform the potential participant about the study, and assess eligibility.

9b. Informed Consent

Written informed consent will be sought from eligible clients who initiated PrEP and have an HIV positive rapid test to collect blood specimens for HIV drug resistance testing and drug level testing. The informed consent process and form will comply with ICH GCP regulations. The original consent form will be filed in the clinic and a copy of the form will be offered to the client.

Figure 1
Study Procedure Flowchart

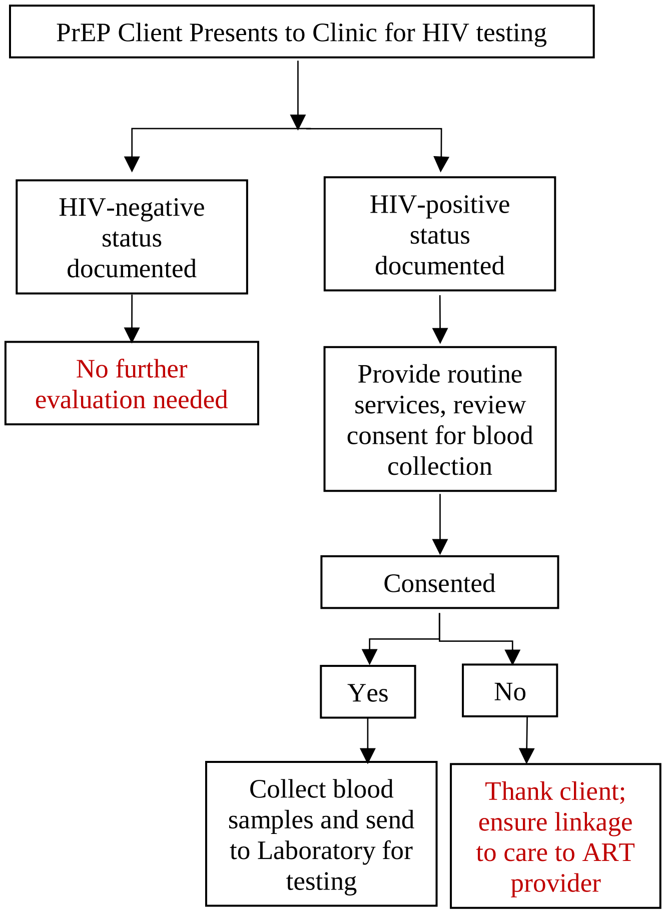

9c. Specimen Collection

After HIV infection is confirmed and the participant consents, the study coordinator, or designee, will collect the blood sample according to standard operating procedures for collection by venipuncture. Depending on site capacity, sites will prepare and ship, either DBS, plasma or whole blood to the testing laboratory. Plasma would be prepared by standard operating procedures at the site. DBS would be prepared by pipetting whole blood onto two DBS cards, of 5 spots each. Once the DBS is collected and dried, all blood samples will be individually packaged and shipped to the testing laboratories. Leftover specimens may be temporarily stored for re-testing due to test failure or quality control.

DBS cards may be stored at ambient temperature for up to 5 days total including the time needed for shipping. Once DBS cards arrive at the laboratories, cards will be stored at -80ºC until they are tested. Whole blood must be shipped at ambient temperature immediately upon collection. If whole blood cannot be shipped the same day as collection, DBS cards should be prepared instead. Plasma must be stored at -80°C. Plasma should not be prepared if storage in a -80°C freezer and shipment on dry ice are unavailable.

Blood collection is expected to be completed at the time of HIV seroconversion confirmation. However, if for some reason the sample collection is not completed on the day of seroconversion confirmation, clients will be asked to return as soon as possible to complete the procedure.

9d. Data Collection

The study coordinator, or designee, will complete the laboratory requisition form [insert other data collection forms as applicable] by reviewing the client's medical file as well as interviewing the client to determine self-reported adherence level.

9e. Laboratory Testing

Drug Resistance Testing

Drug resistance testing will be performed at the [insert] laboratory using one DBS card, or plasma from whole blood, from HIV infected individuals. All information received by the testing laboratories will be unlinked and the laboratory staff will remain blinded to the client apart from gender and date of birth. The sample and results will be tracked only by a sample specific barcode.

[*Update following text based on testing method per laboratory SOP*]. The laboratory will perform the resistance test using a laboratory developed population genotyping assay, per laboratory standard operating procedures. Briefly, a 1.5kb fragment of protease and reverse transcriptase will be reverse transcribed from extracted viral RNA. The amplicon that has been generated will be purified and sequenced using the BigDye® Terminator v3.1 Cycle Sequencing Kit (Applied Biosystems, Foster City, CA), and analyzed on the ABI Prism™ 3500 Genetic Analyzer (Applied Biosystems). The SeqScape software program (Applied Biosystems) will be used to edit the raw sequences and generate consensus sequences. HIV DRMs and drug susceptibility profiles (HIVd algorithm) will be determined using the Stanford University Drug-Resistance Database (Palo Alto, CA). Subtype will be determined using the REGA HIV-1 Subtyping Tool.

Drug Level Testing

[*Update following text based on testing method per laboratory SOP*]. Drug level testing will be performed at the [insert] laboratory using one DBS card or plasma sample. All information received by the testing laboratory will be unlinked and the sample and results will be tracked only by a sample specific barcode. The laboratory will perform the test using a laboratory developed pharmacokinetic assay, per laboratory standard operating procedures. Briefly, tenofovir-diphosphate (TFV-DP) will be quantified by a validated liquid chromatography tandem mass spectrometry assay[21]. TFV-DP steady state concentration (Css) will

Supplement to: Levy L, Peterson J, Kudrick, et al. Casting a wide net: HIV drug resistance monitoring in pre-exposure prophylaxis seroconverters in the Global Evaluation of Microbicide Sensitivity Project. Glob Health Sci Pract. 2022;10(2): e2100122. https://doi.org/10.9745/GHSP-D-21-00122

be determined with a one-compartment first-order model fit to all available concentration data and compared to pharmacologic standards that estimate adherence as high, moderate or low/no adherence.

9f. Provision of test results

Once the resistance test is completed, the applicable laboratory will provide the resistance test results in an easy to understand format to the clinic coordinator or other site staff member. The clinic staff will retrieve the result and confirm client identification as per the barcode sticker. The clinician, or other designee, will either counsel the client on their results and implications for future treatment, or forward the results directly to the client's ART provider upon request.

9. STATISTICAL CONSIDERATIONS

The proportion of drug resistance among the [insert] HIV-infected PrEP clients will be computed along with (exact) 95% confidence intervals (using the Clopper-Pearson method). In addition, frequencies, proportions, and exact 95% confidence intervals of specific mutations (e.g., K65R, K70E, M184I and M184V) will be computed and presented.

Basic socio-demographic data for participating clients will be summarized using the mean, the median, standard deviation, quartiles, and range (minimum and maximum) for continuous variables, proportions for binomial responses, and contingency tables for categorical variables. Logistic regression models will be used to explore differences (e.g., adherence levels, age, and gender) between HIV-positive participants who are drug resistant or not drug resistant if the sample size in each category is sufficient to perform such analyses. Exploratory analyses may be done on resistance data obtained from standard resistance testing to identify polymorphic or subtype-specific sequence changes in HIV-1 that may be associated with ARV resistance.

10. ETHICAL CONSIDERATIONS

The healthcare facilities participating in this study will make efforts to minimize risks of study procedures to participants. Potential risks and benefits include the following:

Risks

Participants may become worried about their privacy and confidentiality. Every effort will be made to protect privacy and confidentiality. Participant visits will take place in private.

Phlebotomy may lead to discomfort or pain, feelings of dizziness or faintness, and/or bruising, swelling, small clot and/or infection.

Benefits

Participants may experience no direct benefit from participation in this study. However, they may appreciate the opportunity to contribute to the field of HIV research. Resistance test results may also

provide important clinical information for the PrEP client and their clinician enabling better ART regimen choice.

Confidentiality

All study-related information will be stored securely at the healthcare facilities. To maintain confidentiality a coded number will identify all blood specimens, reports, and data collection. Materials that link participant barcoded numbers to other identifying information will be stored in an area with limited access.

11. DATA ANALYSIS

After performing the resistance test assay, the applicable laboratory will upload the AB1 files and the data collection forms associated with the resistance test results into a secure Laboratory Information Management System (LIMS). All samples, original files, and forms will remain onsite at the laboratory. Adherence will be measured by detectable drug levels in blood. These de-identified results for both drug resistance and drug levels will be entered into a central database that is maintained by [insert]. [Insert] will conduct a blinded analysis of all results, and present the results and recommendations in reports to project partners.

12. PUBLICATION POLICY AND RESULT DISSEMINATION

Representatives from partner organizations will be invited to co-author any presentation, abstract, or manuscript that utilizes these data. All partner organizations will review and approve all abstracts and manuscripts prior to submission to a conference or journal.

13. REFERENCES

1. UNAIDS. 90-90-90 An ambitious treatment target to help end the AIDS epidemic. http://wwwunaidsorg/sites/default/files/media_asset/90-90-90_en_0pdf. 2014.
2. UNAIDS. *Global AIDS Update.* May 2016.
3. WHO. GUIDELINE ON WHEN TO START ANTIRETROVIRAL THERAPY AND ON PRE-EXPOSURE PROPHYLAXIS FOR HIV. *Geneva, WHO.* 2015, September.
4. Liu AY, Cohen SE, Vittinghoff E, et al. Preexposure Prophylaxis for HIV Infection Integrated With Municipal- and Community-Based Sexual Health Services. *JAMA Intern Med.* 2016;176(1):75-84.
5. Molina JM, Capitant C, Spire B, et al. On-Demand Preexposure Prophylaxis in Men at High Risk for HIV-1 Infection. *N Engl J Med.* 2015;373(23):2237-2246.
6. Baeten JM, Donnell D, Ndase P, et al. Antiretroviral prophylaxis for HIV prevention in heterosexual men and women. *The New England journal of medicine.* 2012;367(5):399-410.
7. Amico KR. Adherence to preexposure chemoprophylaxis: the behavioral bridge from efficacy to effectiveness. *Curr Opin HIV AIDS.* 2012;7(6):542-548.
8. Koss CA, Bacchetti P, Hillier SL, et al. Differences in Cumulative Exposure and Adherence to Tenofovir in the VOICE, iPrEx OLE, and PrEP Demo Studies as Determined via Hair Concentrations. *AIDS Res Hum Retroviruses.* 2017.
9. Van Damme L, Corneli A, Ahmed K, et al. Preexposure prophylaxis for HIV infection among African women. *N Engl J Med.* 2012;367(5):411-422.
10. Grant RM, Lama JR, Anderson PL, et al. Preexposure chemoprophylaxis for HIV prevention in men who have sex with men. *The New England journal of medicine.* 2010;363(27):2587-2599.
11. Thigpen MC, Kebaabetswe PM, Paxton LA, et al. Antiretroviral preexposure prophylaxis for heterosexual HIV transmission in Botswana. *N Engl J Med.* 2012;367(5):423-434.
12. Marrazzo JM, Ramjee G, Richardson BA, et al. Tenofovir-based preexposure prophylaxis for HIV infection among African women. *N Engl J Med.* 2015;372(6):509-518.
13. McCormack S, Dunn DT, Desai M, et al. Pre-exposure prophylaxis to prevent the acquisition of HIV-1 infection (PROUD): effectiveness results from the pilot phase of a pragmatic open-label randomised trial. *Lancet.* 2016;387(10013):53-60.
14. Fonner VA, Dalglish SL, Kennedy CE, et al. Effectiveness and safety of oral HIV preexposure prophylaxis for all populations. *AIDS.* 2016;30(12):1973-1983.
15. Parikh UM, Mellors JW. Should we fear resistance from tenofovir/emtricitabine preexposure prophylaxis? *Curr Opin HIV AIDS.* 2016;11(1):49-55.
16. Clutter DS, Jordan MR, Bertagnolio S, Shafer RW. HIV-1 drug resistance and resistance testing. *Infection, genetics and evolution : journal of molecular epidemiology and evolutionary genetics in infectious diseases.* 2016;46:292-307.
17. Nishimura Y, Martin MA. The acute HIV infection: implications for intervention, prevention and development of an effective AIDS vaccine. *Curr Opin Virol.* 2011;1(3):204-210.
18. Sanders EJ, Mugo P, Prins HA, et al. Acute HIV-1 infection is as common as malaria in young febrile adults seeking care in coastal Kenya. *AIDS.* 2014;28(9):1357-1363.
19. Bebell LM, Pilcher CD, Dorsey G, et al. Acute HIV-1 infection is highly prevalent in Ugandan adults with suspected malaria. *AIDS.* 2010;24(12):1945-1952.
20. Group ISS, Lundgren JD, Babiker AG, et al. Initiation of Antiretroviral Therapy in Early Asymptomatic HIV Infection. *N Engl J Med.* 2015;373(9):795-807.

21. Castillo-Mancilla JR, Zheng JH, Rower JE, et al. Tenofovir, emtricitabine, and tenofovir diphosphate in dried blood spots for determining recent and cumulative drug exposure. *AIDS Res Hum Retroviruses.* 2013;29(2):384-390.

Supplement to: Levy L, Peterson J, Kudrick, et al. Casting a wide net: HIV drug resistance monitoring in pre-exposure prophylaxis seroconverters in the Global Evaluation of Microbicide Sensitivity Project. Glob Health Sci Pract. 2022;10(2): e2100122. https://doi.org/10.9745/GHSP-D-21-00122

Appendix I
Template Informed Consent Form (English)

Title: **An assessment of the Risk of HIV Drug Resistance among PrEP Users**
Funded by: [insert]
Project Partners: [insert]

Introduction: My name is _______ and I am talking to all people who became infected with HIV while using pre-exposure prophylaxis (otherwise known as PrEP). We want to better understand why you became infected with HIV. We also want to know if your HIV virus has developed drug resistance. Drug resistance is when one or more medicines that usually work to treat HIV, called antiretrovirals or ARVs, no longer work as well.

Information about the project: This project will take place in different health facilities that are providing PrEP in [insert country]. We expect to enroll about [insert number] people across all the health facilities.

Your role in the project: We would like to take approximately 10 ml (about two teaspoonfuls) of blood from you at the time that you have a positive HIV test. We will also be asking a few questions about you, such as your age and how often you have been using PrEP up until this point. We may also look for this information in the records that the health center already collects. If you agree to this blood test, we will check for drug resistance in your blood and then let you know when the results are ready to share with you or your doctor. We will also check for the amount of drug in your blood, but this information will not be shared with you because it is for research purposes only and not important for your medical care. Lab testing for this study will be done in [insert countries as needed].

Possible risks: There is minimal risk in this project, but it is possible that there could be discomfort, or very rarely an infection, from the blood draw. There also is the risk that others may learn that you participated in this project. Every effort will be made to keep information about you safe; however, this cannot be guaranteed.

Possible benefits: You will find out if you have any drug resistance and this information can be used by your doctors to know which ARV medication might work best for you. You may also get some personal satisfaction from helping us understand if people who get HIV while using PrEP may have drug resistance.

If you decide to not participate: You are free to participate or not in this study. If you decide not to participate, you can continue to get your healthcare at this facility or other facilities, but we will not be able to take your blood for resistance testing. If you decide to participate but later change your mind, you can, and your rights to receive services at health centers will not be affected.

Confidentiality: Your name will not be recorded in the computer that we will use to look at your drug resistance results. Your name will not appear in any reports or publications. All information collected for this project will be kept in a locked cabinet or room.

Compensation: Your participation is voluntary; no monetary or other compensation will be given.

If you have questions, you can call: [insert name/phone number]

Volunteer Agreement: If you have read this consent form (or had it explained to you), all your questions have been answered and you agree to take part in this project, please sign your name below.

Client's Name (Print)	Client's Signature	Date
Clinic Staff Conducting Consent Process Name (print)	Clinic Staff Signature	Date
Witness Name	Witness Signature	Date
